# Adaptation effects in static postural control by providing simultaneous visual feedback of center of pressure and center of gravity

**DOI:** 10.1186/s40101-017-0147-5

**Published:** 2017-07-19

**Authors:** Kenta Takeda, Hiroki Mani, Naoya Hasegawa, Yuki Sato, Shintaro Tanaka, Hiroshi Maejima, Tadayoshi Asaka

**Affiliations:** 1 Graduate School of Health Sciences, Hokkaido University, N12-W5, Kita-ku, Sapporo, Hokkaido 060-0812 Japan; 2 Department of Rehabilitation Science, Faculty of Health Sciences, Hokkaido University, N12-W5, Kita-ku, Sapporo, Hokkaido 060-0812 Japan

**Keywords:** Center of gravity, Center of mass, Center of pressure, Static posture, Visual feedback training

## Abstract

**Background:**

The benefit of visual feedback of the center of pressure (COP) on quiet standing is still debatable. This study aimed to investigate the adaptation effects of visual feedback training using both the COP and center of gravity (COG) during quiet standing.

**Methods:**

Thirty-four healthy young adults were divided into three groups randomly (COP + COG, COP, and control groups). A force plate was used to calculate the coordinates of the COP in the anteroposterior (COP_AP_) and mediolateral (COP_ML_) directions. A motion analysis system was used to calculate the coordinates of the center of mass (COM) in both directions (COM_AP_ and COM_ML_). The coordinates of the COG in the AP direction (COG_AP_) were obtained from the force plate signals. Augmented visual feedback was presented on a screen in the form of fluctuation circles in the vertical direction that moved upward as the COP_AP_ and/or COG_AP_ moved forward and vice versa. The COP + COG group received the real-time COP_AP_ and COG_AP_ feedback simultaneously, whereas the COP group received the real-time COP_AP_ feedback only. The control group received no visual feedback. In the training session, the COP + COG group was required to maintain an even distance between the COP_AP_ and COG_AP_ and reduce the COG_AP_ fluctuation, whereas the COP group was required to reduce the COP_AP_ fluctuation while standing on a foam pad. In test sessions, participants were instructed to keep their standing posture as quiet as possible on the foam pad before (pre-session) and after (post-session) the training sessions.

**Results:**

In the post-session, the velocity and root mean square of COM_AP_ in the COP + COG group were lower than those in the control group. In addition, the absolute value of the sum of the COP − COM distances in the COP + COG group was lower than that in the COP group. Furthermore, positive correlations were found between the COM_AP_ velocity and COP − COM parameters.

**Conclusions:**

The results suggest that the novel visual feedback training that incorporates the COP_AP_–COG_AP_ interaction reduces postural sway better than the training using the COP_AP_ alone during quiet standing. That is, even COP_AP_ fluctuation around the COG_AP_ would be effective in reducing the COM_AP_ velocity.

## Introduction

The ability to maintain balance in static postures relies on the ability of the central nervous system to control movements or positional fluctuations by using the body’s center of mass (COM) so that it remains within safe boundaries above the base of support [1, 2]. If static balance during quiet standing is controlled by shifting the center of pressure (COP) through the feet, the body will move as a single segment, often modeled as an inverted pendulum [3]. Previous studies have reported that the augmented visual feedback of the COP has been used for static balance training [4–6].

However, the benefits gained from visual feedback of the COP during quiet standing are still under debate [7, 8]. Kilby et al. reported that the real-time visual feedback of neither the COP nor the COM affected the postural motion of healthy adults during quiet standing [9]; in other words, neither the COP nor the COM velocities changed when conditions were altered between a presence and lack of augmented visual feedback. In addition, the participants in the study of Lakhani et al. showed no postural stability learning effects when visual feedback training was using either as a vertical projection of the COM onto the ground (i.e., the center of gravity (COG)) or the COP during quiet standing [10]. In fact, under no feedback conditions did the root mean square values of the COP or COG change between the pre-training and post-training sessions.

The impact of the difference in position between the COP and COG (COP–COG distance) on postural stability in static balance has also been investigated [11]. A larger COP–COG distance has been shown to indicate greater body acceleration during quiet standing [12, 13]. During quiet standing, the COP–COG distance increases with age and depends on whether the participant’s eyes are closed or open [12, 14]. Postural instability may result from biased positioning of the COP relative to the COG, as this will result in a unidirectional moment acting on the COM [3]. In fact, Mani et al. reported that elderly participants were not able to maintain equilibrium standing on one leg when the position of the COP was biased relative to the COG, such as when the COP was located laterally in the direction of the supporting leg, while the younger group of participants experienced less difficulty [14]. In addition, Ibuki et al. reported that the COP − COM distance decreased and the COP fluctuated more evenly around the COM during one-legged standing in the ballet dancer group than in the control group [15]. These findings imply that feedback training incorporating the interaction between the COP and COG may be more effective for improving static balance than training using the COP or COG alone.

With this background in mind, the purpose of this study was to investigate the adaptive effects of augmented visual feedback training using both the COP and COG, as compared to training using only the COP, during quiet standing. The hypothesis was that novel balance training, which incorporates the interaction between the COP and COG using simultaneously the visual feedback of both, would reduce postural sway compared to training that used the COP alone without feedback. The findings of this study could contribute toward the development of an effective visual feedback training system for improving postural static balance.

## Methods

### Participants

Thirty-four healthy young adults without any known neurological, motor, or visual disorders or disabilities participated in this study. All the study protocols were approved by the ethics committee of the institution where the study was conducted, and written informed consent was obtained from all participants according to the Declaration of Helsinki. The age, sex, height, body weight, and foot length of each participant were recorded (Table 1). The participants were divided randomly into three groups. The first group, i.e., the COP group, received visual feedback of their real-time COP during the training session. The second group, i.e., the COP + COG group, received real-time visual feedback of their COG as well as their COP during the training session. The third group, i.e., the control group, received no visual feedback and was instructed to focus on a fixed visual target.

**Table 1:** The characteristics of the COP, COP + COG, and control groups

	COP (*n* = 11)	COP + COG (*n* = 12)	Control (*n* = 11)
Age (years)	23.2 ± 2.3	22.8 ± 1.6	22.3 ± 2.4
Sex	Male 6, female 5	Male 8, female 4	Male 5, female 6
Height (cm)	165.7 ± 6.9	169.2 ± 7.6	164.5 ± 8.3
Body weight (kg)	56.8 ± 7.5	59.7 ± 9.0	57.0 ± 9.2
Foot length (cm)	24.2 ± 1.8	24.5 ± 1.6	23.2 ± 1.7

Mean ± SD

### Equipment

Kinematic data were collected using a six-camera 3D motion analysis system (Motion Analysis Corporation, Santa Rosa, CA, USA) at a sampling frequency of 200 Hz. Twenty reflective markers were attached to the following bony landmarks: the acromioclavicular joint, the lateral epicondyle of the upper arm, the wrist, the head of the second metacarpal, the great trochanter of the femur, the lateral malleolus, the second metatarsal head, the calcaneus, and the C7, S1, and bilateral point of the external acoustic foramen. These markers were used to calculate the COM in the anteroposterior (AP) and mediolateral (ML) directions (COM_AP_ and COM_ML_), based on the 14 body segments and an anthropometrical model [3]. A force plate (Kistler, Winterthur, Switzerland) was used to calculate the coordinates of the COP in the AP (COP_AP_; Appendix 1) and ML (COP_ML_) directions. Force plate signals were collected at a sampling frequency of 1000 Hz and synchronized with the motion analysis system. The real-time COG in the AP direction (COG_AP_) was obtained from the force plate signals (Appendix 2) [10, 12].

Augmented visual feedback was provided in the form of fluctuating circles moving vertically upward as the COP_AP_ and COG_AP_ moved forward and downward as they moved backward. LabVIEW software (National Instruments, USA) was used to present this feedback on a screen (height 1.8 m, width 2.5 m) located approximately 5 m away from the participant. The vertical movement of the circles on the screen was 16 times greater than the true COP_AP_ and COG_AP_ displacements [16].

### Procedures

The participants stood with both their feet placed on a foam pad (thickness 6.5 cm, SAKAI Medical, Japan) throughout the pre-training, training, and post-training sessions. Only the AP direction was applied to reduce feedback complexity and allow the participants to focus on minimizing COP and COG fluctuations along a single axis [10]. The distance of the two horizontal lines on the screen corresponded to the two standard deviations (SD) of the first COG_AP_ displacement measurement. The center point between the two lines identified the center of the force plate in the AP direction.

**Fig. 1:**
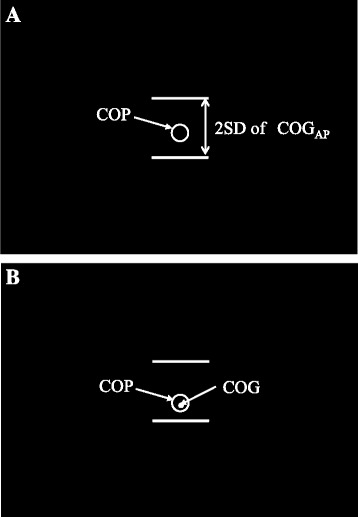
Visual feedback on screen for the **a** COP group and **b** COP + COG group. *Blank circles* represent the real-time COP_AP_, and *filled circle* represent the real-time COG_AP_. These *circles* move in the vertical direction 16 times greater than the real amount of COP_AP_ or COG_AP_ movements. The width between the two *horizontal lines* indicates each participant’s 2SD of COG_AP_ displacements on a force plate during quiet standing

In the pre- and post-training test sessions (hereafter called pre-session and post-session, respectively), participants were required to lock their eyes on the fixed visual target and stand as steadily as possible for 60 s. In the training session, participants in the COP group were required to align the center of the blank circle (φ13.5 cm), which represented their COP_AP_, to the center of the two horizontal lines for 40 s (Fig. 1a). The participants in the COP + COG group were required to align the center of the filled circle to the center of the horizontal lines and maintain an even distance in the up-down direction between the blank and filled circles (φ4.5 cm), which respectively represented their COP_AP_ and COG_AP_, for 40 s (Fig. 1b). In practice, this required the participants to keep the filled circle inside the blank circle. The participants in the control group were instructed to lock their eyes on a fixed visual target and stand as steadily as possible for 40 s.

The participants were instructed to stand barefoot with their arms across their chest in front of a visual target located at an eye-level height on the screen. First, to measure the SD of the COG_AP_ displacements, each participant was instructed to stand quietly with both feet placed together on the force plate with their eyes open for 10 s. Horizontal lines, indicating the two SDs of each participant, were also projected on the screen during the training session (Fig. 1). Subsequently, the participants were required to stand on the foam pad, which was attached to the force plate with double-sided adhesive tapes, as steadily as possible with their feet placed together. The position of the feet on the pad was standardized: the center of the force plate in the sagittal plane was matched with the position of the feet 40% down the length from the heel [17]. The exact location where the feet were to be placed was marked on the pad to ensure that all the participants started with the same foot position in each trial. Each participant was asked to perform 12 trials with a 5-min rest after the first six trials. The break between trials was approximately 1 min, while the time between the trials and pre- or post-sessions was 5 min.

### Data and statistical analysis

All signals were processed offline using MATLAB software (MathWorks, Natick, MA, USA). The motion analysis system data were filtered with a fourth-order 20-Hz low-pass zero-lag Butterworth filter, and the force plate data were filtered with a fourth-order 8-Hz low-pass zero-lag Butterworth filter. Although the signals obtained in the pre- and post-sessions had a duration of 60 s, only 50-s durations of the signals were analyzed, excluding the initial and final 5 s. The mean absolute velocities and root mean squares of both the COP and COM in the AP and ML directions (COP_AP/ML_ velocity and COM_AP/ML_ velocity, COP_AP/ML_ RMS and COM_AP/ML_ RMS) were calculated to assess postural stability. Furthermore, the mean absolute value of the COP − COM distance (COP − COM_close_, Eq. 1) and the absolute value of the sum of the COP − COM distance (COP − COM_even_, Eq. 2) in the AP direction were calculated as follows:

1 $$ \mathrm{COP}-{\mathrm{COM}}_{\mathrm{close}}=\frac{{\displaystyle {\sum}_{t=1}^N\left|{\mathrm{COP}}_t-{\mathrm{COM}}_t\right|}}{N} $$

2 $$ \mathrm{COP}-{\mathrm{COM}}_{\mathrm{even}}=\left|{\displaystyle {\sum}_{t=1}^N\left({\mathrm{COP}}_t-{\mathrm{COM}}_t\right)}\right| $$

where *N* is the total sampling number. Thus, a lower value of COP − COM_close_ indicated that movements of the COP were held closer to the COM in the AP direction. A shorter COP − COM_even_ indicated that the COP position was more even, with fewer fluctuations around the COM in the AP direction. All parameters were normalized by the foot length (FL) of each participant.

Both one-way and two-way mixed-design ANOVA were used in each group (factor *Group*: COP, COP + COG, and control); one-way ANOVA was used to identify and analyze differences in biomechanical characteristics, and two-way mixed-design ANOVA compared the group data to test sessions (factor *Test Session*: pre and post) to analyze possible differences in the value of the indices. A post hoc analysis was performed using Bonferroni pairwise comparison, and Pearson’s correlation coefficient was used to identify and analyze correlations between the COM_AP_ velocity and COP − COM parameters. The statistical significance was set to *p* < 0.05 for all tests.

## Results

No significant differences were observed among the three groups in terms of age (*F*_2, 31_ = 0.500, *p* = 0.611), height (*F*_2, 31_ = 1.170, *p* = 0.324), weight (*F*_2, 31_ = 0.391, *p* = 0.680), and foot length (*F*_2, 31_ = 1.779, *p* = 0.186) (Table 1).

**Fig. 2:**
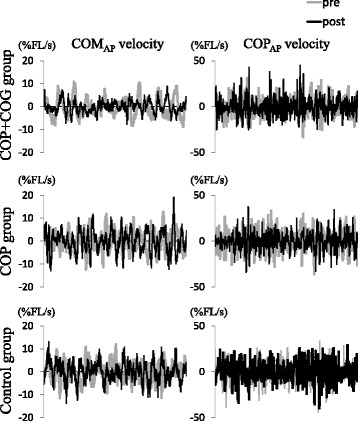
The typical data of COM_AP_ and COP_AP_ velocity profiles. The data of the COP + COG, COP, and control groups are represented from the *top* to *bottom columns*, respectively. The *gray lines* represent velocities during the pre-test session, and the *black lines* represent those during the post-test session

Figure 2 shows the characteristic data of COM_AP_ and COP_AP_ velocity profiles in the pre- and post-sessions for each group. In the COP + COG group, the amplitude of the COM_AP_ velocity during the post-session decreased compared to the pre-session values, while the amplitude of the COP_AP_ velocity did not change. Table 2 lists the mean and SD of velocities and RMSs of both the COM and COP in the AP and ML directions.

**Table 2:** The results of postural stability

	COP + COG		COP		Control	
	Pre	Post	Pre	Post	Pre	Post
COM_AP_ velocity (%FL/s)	2.91 ± 0.52*	2.27 ± 0.41^†^	2.94 ± 0.58	2.74 ± 0.66	3.02 ± 0.41*	2.61 ± 0.34
COM_AP_ RMS (%FL)	3.24 ± 0.72	2.92 ± 0.86^†^	3.38 ± 1.21	2.86 ± 0.62	3.80 ± 1.09	4.01 ± 1.25
COP_AP_ velocity (%FL/s)	8.40 ± 2.06	8.39 ± 1.52	9.26 ± 2.97	9.07 ± 3.57	8.95 ± 1.66	7.86 ± 1.75
COP_AP_ RMS (%FL)	3.96 ± 0.73	3.54 ± 0.78	4.12 ± 1.13	3.54 ± 0.64	4.33 ± 0.95	4.33 ± 1.12
COM_ML_ velocity (%FL/s)	2.99 ± 0.52	2.76 ± 0.34	3.40 ± 0.41*	3.12 ± 0.59	3.10 ± 0.70	2.78 ± 0.38
COM_ML_ RMS (%FL)	2.54 ± 0.44	2.59 ± 0.49	2.54 ± 0.40*	3.11 ± 0.67	2.27 ± 0.38	2.59 ± 0.60
COP_ML_ velocity (%FL/s)	9.12 ± 2.51	7.99 ± 1.24	10.48 ± 3.02*	8.85 ± 2.72	9.48 ± 2.59	8.63 ± 1.85
COP_ML_ RMS (%FL)	3.43 ± 0.47	3.33 ± 0.46	3.61 ± 0.44	3.81 ± 0.74	3.25 ± 0.54	3.29 ± 0.60

Mean ± SD

*p < 0.05, between test sessions

^†^ *p* < 0.05, compared to that of the control group in the post-session

No significant effect was observed on the COM_AP_ velocity between *Group* factors (*F*_2, 31_ = 0.946, *p* = 0.399). However, the COM_AP_ velocity showed a significant change between *Test session* factors (*F*_1, 31_ = 42.361, *p* < 0.001). A significant interaction was observed between the *Group* and *Test session* factors in terms of the COM_AP_ velocity (*F*_2, 31_ = 3.391, *p* = 0.047). The post hoc test revealed that post-session COM_AP_ velocity values were significantly lower in the COP + COG group compared to those in the control group (*p* = 0.047). For both the COP + COG and control groups, the COM_AP_ velocity in the post-session was significantly lower than that in the pre-session (*p* < 0.001); however, no significant difference was observed in the COP group between the pre- and post-sessions (*p* = 0.117).

In terms of COM_AP_ RMS, no significant main effect was observed for the factor *Test session* (*F*_1, 31_ = 0.979, *p* = 0.330), nor was there a significant interaction (*F*_2, 31_ = 1.036, *p* = 0.367). On the other hand, the COM_AP_ RMS showed a significant main effect for the factor *Group* (*F*_2, 31_ = 4.158, *p* = 0.025). The post hoc test revealed that the overall COM_AP_ RMS of the COP + COG group was significantly lower than that of the control group (*p* = 0.042), while there was no significant difference between that of the COP and control groups (*p* = 0.068). The post-session COM_AP_ RMS of the COP + COG group was significantly lower than that of the control group (*p* = 0.022); however, there was no significant difference in the pre-session (*p* = 0.154).

In terms of the COP_AP_ velocity and COP_AP_ RMS, no significant differences were observed between the *Group* (COP_AP_ velocity *F*_2, 31_ = 0.443, *p* = 0.646; COP_AP_ RMS *F*_2, 31_ = 2.199, *p* = 0.128) and *Test session* factors (COP_AP_ velocity *F*_1, 31_ = 2.544, *p* = 0.121; COP_AP_ RMS *F*_1, 31_ = 3.106, *p* = 0.088). No significant interaction (COP_AP_ velocity *F*_2, 31_ = 1.520, *p* = 0.235; COP_AP_ RMS *F*_2, 31_ = 0.797, *p* = 0.460; Table 2) was observed.

With regard to COM_ML_ velocity and COM_ML_ RMS, no significant main effect was observed for the factor *Group* (COM_ML_ velocity *F*_2, 31_ = 2.329, *p* = 0.114; COM_ML_ RMS *F*_2, 31_ = 2.842, *p* = 0.074), nor was there a significant interaction (COM_ML_ velocity *F*_2, 31_ = 0.121, *p* = 0.887; COM_ML_ RMS *F*_2, 31_ = 2.049, *p* = 0.146). In contrast, the COM_ML_ velocity and COM_ML_ RMS showed significant main effects for the factor *Test session* (COM_ML_ velocity *F*_1, 31_ = 12.497, *p* = 0.001; COM_ML_ RMS *F*_1, 31_ = 8.57, *p* = 0.006). The post hoc test revealed that the post-session COM_ML_ velocity of the COP group was significantly lower than that for the pre-session (*p* = 0.04). However, in the COP + COG and control groups, no significant difference was observed between the sessions (COP + COG group, *p* = 0.125; control group, *p* = 0.056). Meanwhile, the post-session COM_ML_ RMS of the COP group was significantly higher than that for the pre-session (*p* = 0.009). The COM_ML_ RMS of the COP + COG and control groups showed no significant difference between the sessions (COP + COG group, *p* = 0.82; control group, *p* = 0.114).

With regard to COP_ML_ velocity, no significant main effect was observed for the factor *Group* (*F*_2, 31_ = 0.722, *p* = 0.494), nor was there a significant interaction (*F*_2, 31_ = 0.526, *p* = 0.596). On the contrary, the COP_ML_ velocity presented a significant main effect for the factor *Test session* (*F*_1, 31_ = 15.86, *p* < 0.001). The post hoc test revealed that the post-session COP_ML_ velocity of the COP group was significantly lower than that for the pre-session (*p* < 0.001). However, in the COP + COG and control groups, no significant difference was found between the sessions (COP + COG group, *p* = 0.081; control group, *p* = 0.195).

Meanwhile, the COP_ML_ RMS demonstrated no significant differences between the *Group* (*F*_2, 31_ = 2.697, *p* = 0.083) and *Test session* factors (*F*_1, 31_ = 0.24, *p* = 0.628). No significant interaction (*F*_2, 31_ = 0.789, *p* = 0.463) was observed.

**Fig. 3:**
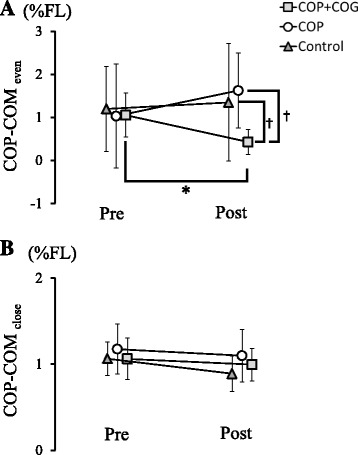
Mean ± SD of **a** COP − COM_even_ and **b** COP − COM_close_ for both groups in pre- and post-sessions. The *white circles* represent the COP group, the *gray squares* represent the COP + COG group, and the *dark gray triangles* represent the control group. COP − COM_even_ for the COP + COG group is seen to be significantly smaller than that for the COP group in the post-session (*p < 0.05). For the COP + COG group, COP − COM_even_ in the post-session was significantly smaller than that in the pre-session (*p < 0.05). The data were normalized by the foot length (FL) for each participant

No significant differences were observed between the *Group* (*F*_2, 31_ = 2.162, *p* = 0.132) or *Test session* factors (*F*_1, 31_ = 0.047, *p* = 0.829) in terms of COP − COM_even_. A significant COP − COM_even_ interaction was observed between the two factors (*F*_2, 31_ = 3.517, *p* = 0.042; Fig. 3a): The post hoc test revealed that the post-session COP − COM_even_ was significantly smaller for the COP + COG group than that for the pre-session (*p* = 0.002). On the other hand, no significant difference was observed between the pre- and post-sessions in the COP group (*p* = 0.234). Furthermore, the COP − COM_even_ for the COP + COG group was significantly smaller than that for the COP group (*p* < 0.001) or control group (*p* = 0.033) in the post-session.

In terms of COP − COM_close_, no significant effects were observed for the factor *Group* (*F*_2, 31_ = 1.457, *p* = 0.248) and no significant interaction (*F*_2, 31_ = 1.267, *p* = 0.296) was observed. On the other hand, the factor *Test session* showed significant discrepancies (*F*_1, 31_ = 11.884, *p* = 0.002; Fig. 3b).

**Fig. 4:**
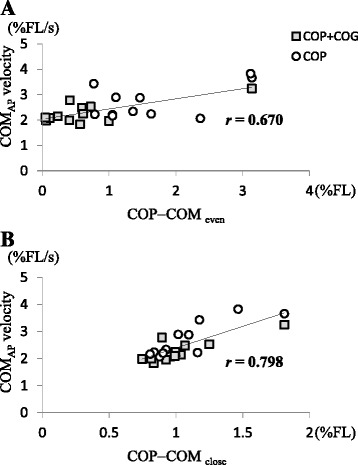
Relationship between the COM_AP_ velocity, **a** COP − COM_even_, and **b** COP − COM_close_. A significant positive correlation was observed between the COM_AP_ velocity and COP − COM_even_ (*r* = 0.670) and COP − COM_close_ (*r* = 0.789). The data for COP + COG and COP groups were combined. The abbreviations are the same as those used in Fig. 3

In the post-session, significant positive correlations were observed between the COM velocity and COP − COM_even_ in the COP + COG group (*r* = 0.729, *p* < 0.01) and the COP group (*r* = 0.526, *p* < 0.05). Significant positive correlations were observed between the COM velocity and COP − COM_close_ in the COP + COG group (*r* = 0.823, *p* < 0.01) and the COP group (*r* = 0.843, *p* < 0.01). No significant correlations were observed between the COM velocity and COP − COM_even_ (*r* = −0.291, *p* > 0.05) or COP − COM_close_ (*r* = 0.457, *p* > 0.05) in the control group. Significant positive correlations were observed between the COM velocity and COP − COM_even_ (*r* = 0.670, *p* < 0.01: Fig. 4a) as well as COP − COM_close_ (*r* = 0.798, *p* < 0.01: Fig. 4b) for the combined data of the COP + COG and COP groups.

## Discussion

The mean absolute velocity of the COM has been proven to be a highly reliable and sensitive indicator of postural sway [18, 19]. The main finding of this study is that the COM_AP_ velocity decreased after the training session in the COP + COG and control groups, but not in the COP group. The COM_AP_ velocity in the COP + COG group was lower than that in the control group during post-session quiet standing (Table 2). Furthermore, the COM_AP_ RMS was significantly lower in the COP + COG group compared to that in the control group following training. These results suggest that training conducted using simultaneous COP_AP_ and COG_AP_ visual feedback increases postural stability compared to training using the COP_AP_ alone under a no-feedback condition. Therefore, the results of this study have confirmed the hypothesis.

The effects that were related to a decrease in the post-session COM_AP_ velocity would be enhanced by even fluctuations of the COP_AP_ around the COG_AP_ (Fig. 3a) because of the significant correlation between the COM_AP_ velocity and COP − COM_even_ (Fig. 4a). According to the inverted pendulum model, inertial forces produced by even fluctuations of the COP toward the COG restrain COM movements toward the center of its fluctuation range; this is because the COP − COM distance reflects the moment arm for inertial forces, such as propulsion toward the COM or braking against movements toward the COM [20, 21].

Interestingly, the COP_AP_ velocity did not decrease even though the COM_AP_ velocity decreased in the post-session for each group (Table 2). In general, minimizing the COM displacement would be expected to result in a concurrent decrease in the COP displacement [22]. However, Carpenter et al. [23] and Murnaghan et al. [24] showed that COP displacements increased when COM movements were stabilized. They proposed that COP fluctuations played an exploratory role, gathering sensory information during quiet standing. The results of this study indicate that a decrease in the COM_AP_ velocity do not result in a concurrent decrease in the COP_AP_ velocity; as such, the COP and COM velocities may realistically behave in different ways.

No significant differences of the velocity and the RMS of COM and COP between the pre- and post-sessions were found in the COP + COG group in the ML direction. Therefore, the decreased postural stability in the ML direction after the training could not be confirmed in the COP + COG group. Interestingly, the COM_ML_ and COP_ML_ velocities in the COP group decreased after the training. However, the increased postural stability in the ML direction could not be confirmed because the COM_ML_ RMS in the COP group increased after the training. The standing postural controls for the AP or ML direction are involved in two distinct ankle and hip mechanisms [3]. The possibility effects of the postural control during quiet standing between the two mechanisms by the feedback training should be further investigated in future studies [25].

We suspect that efforts to maintain the COP_AP_ at an even distance from the COG_AP_ may have indirectly contributed to reducing the COP − COM distance; however, no interaction was observed in terms of COP − COM_close_. The quantitative results of this study showed that the COM_AP_ velocity was correlated to COP − COM_close_ (Fig. 4b). Therefore, adding visual targets indicating the COP_AP_ and COG_AP_ (e.g., two other horizontal lines positioned along the centers of the blank circle representing the COP_AP_ and the filled circle representing the COG_AP_) and requiring participants to reduce the distance between these two horizontal lines may also be effective to decelerate the COM_AP_ velocity.

The limitation of this study is that experiments were performed with a small sample size of participants. In addition, the adaptation effects of training may not be detected sufficiently with the small amount of training the participants underwent. Therefore, the training effects in the COP group may not be detected, although the COM_AP_ velocity in the control group decreased after training. Furthermore, the force under the feet may not be identical to that under the foam pad because the force or moment could spread in the pad. The learning effects of this novel balance training should be further investigated with a retention test and applied to individuals with postural instability in future studies.

## Conclusion

Simultaneous visual feedback training that uses both the COP_AP_ and COG_AP_, and focuses on their interaction, reduces postural sway during quiet standing better than the training designed to affect only the COP_AP_ under the no-feedback condition. It can therefore be stated that even COP_AP_ fluctuations around the COG_AP_ would be effective for maintaining postural static balance through an associated reduction in COM_AP_ velocity.

## Appendix 1

The COP_AP_ was calculated as follows:

3 $$ {\mathrm{COP}}_{\mathrm{AP}}=\frac{Fy\times \left(-{D}_0-{D}_{\mathrm{mat}}\right)+ Mx}{Fz} $$

where *Fy* and *Fz* denote the force in the AP and vertical directions, respectively. *Mx* denotes the moment around the lateral axis. *D*_0_ and *D*_mat_ denote the thickness of the force plate and the foam pad, respectively.

## Appendix 2

If human posture during quiet standing can be approximated as an inverted pendulum, the equation of motion can be described as follows:

4 $$ I\ddot{\theta}= m g h\kern0.5em  \sin \kern0.5em \theta - T $$

where *m* is the mass of the body excluding feet, *g* is the gravitational acceleration coefficient, *h* is the distance between COM and the ankle joint, *I* is the moment of inertia of the body about the ankle joint, *θ* is the body angle to vertical axis, $\ddot{\theta}$ is the body angular acceleration, and *T* is the ankle torque produced about the participant’s ankle joint. Assuming that the body sway amplitude is small, *T* ≈ *mg*COP and *mgh* sin *θ mg*COG. Thus, the right-hand side of Eq. (3) can be rewritten as − *mg*(COP − COG). Considering an additional approximation of $\ddot{\theta}\approx C\ddot{O} M/ h= A C C/ h={F}_Y/ m h$, Eq. (3) can be derived as follows:

5 $$ {\mathrm{COG}}_{\mathrm{AP}}\kern0.5em =\kern0.5em {\mathrm{COP}}_{\mathrm{AP}}-\left(\frac{1}{m gh}\right)\ast \frac{Fy}{m\hbox{'}} $$

where *Fy* is the force in the AP direction.

6 $$ m=0.971\mathrm{M}, $$

7 $$ h = 0.547\mathrm{H}, $$

8 $$ I=0.319{\mathrm{MH}}^2, $$

where M is the participant mass of body in kilograms and H is the participant height in meters.

The details of the derivation steps and the anthropometric estimation of body dimensions can be obtained from previous reports [3, 12].
